# Microbial Transformation of Biomacromolecules in a Membrane Bioreactor: Implications for Membrane Fouling Investigation

**DOI:** 10.1371/journal.pone.0042270

**Published:** 2012-08-09

**Authors:** Zhongbo Zhou, Fangang Meng, So-Ryong Chae, Guocheng Huang, Wenjie Fu, Xiaoshan Jia, Shiyu Li, Guang-Hao Chen

**Affiliations:** 1 SYSU-HKUST Research Center for Innovative Environmental Technology (SHRCI ET), School of Environmental Science and Engineering, Sun Yat-sen University, Guangzhou, China; 2 Guangdong Provincial Key Laboratory of Environmental Pollution Control and Remediation Technology, Guangzhou, China; 3 School of Chemical and Biomolecular Engineering, The University of Sydney, New South Wales, Australia; 4 Department of Civil and Environmental Engineering, The Hong Kong University of Science and Technology, Clear Water Bay, Hong Kong, China; Montana State University, United States of America

## Abstract

**Background:**

The complex characteristics and unclear biological fate of biomacromolecules (BMM), including colloidal and soluble microbial products (SMP), extracellular polymeric substances (EPS) and membrane surface foulants (MSF), are crucial factors that limit our understanding of membrane fouling in membrane bioreactors (MBRs).

**Findings:**

In this study, the microbial transformation of BMM was investigated in a lab-scale MBR by well-controlled bioassay tests. The results of experimental measurements and mathematical modeling show that SMP, EPS, and MSF had different biodegradation behaviors and kinetic models. Based on the multi-exponential G models, SMP were mainly composed of slowly biodegradable polysaccharides (PS), proteins (PN), and non-biodegradable humic substances (HS). In contrast, EPS contained a large number of readily biodegradable PN, slowly biodegradable PS and HS. MSF were dominated by slowly biodegradable PS, which had a degradation rate constant similar to that of SMP-PS, while degradation behaviors of MSF-PN and MSF-HS were much more similar to those of EPS-PN and EPS-HS, respectively. In addition, the large-molecular weight (MW) compounds (>100 kDa) in BMM were found to have a faster microbial transformation rate compared to the small-MW compounds (<5 kDa). The parallel factor (PARAFAC) modeling of three-dimensional fluorescence excitation-emission matrix (EEM) spectra showed that the tryptophan-like PN were one of the major fractions in the BMM and they were more readily biodegradable than the HS. Besides microbial mineralization, humification and hydrolysis could be viewed as two important biotransformation mechanisms of large-MW compounds during the biodegradation process.

**Significance:**

The results of this work can aid in tracking the origin of membrane foulants from the perspective of the biotransformation behaviors of SMP, EPS, and MSF.

## Introduction

Biomacromolecules (BMM) are a pool of complex organic compounds, including colloidal and soluble microbial products (SMP) and extracellular polymeric substances (EPS), which are known as important membrane foulants in membrane bioreactors (MBRs) [1], [2]. During the operation of MBRs, bio-cake layers may gradually form as a result of the retention and deposition of BMM and sludge flocs [3]. The BMM in bio-cake layers or gel layers are referred to as membrane surface foulants (MSF). It is known that the production and biodegradation of BMM are ubiquitous in wastewater treatment systems due to the ceaseless microbial metabolism. EPS can be transformed to be SMP by microbial hydrolysis and shear-induced erosion [4]. In MBRs, the longer sludge retention time (SRT) and the higher aeration intensity can impact EPS release [5], [6]. Furthermore, the readily biodegradable SMP can be quickly degraded by microorganisms [7]. As such, the refractory and large-size BMM accumulated in the bioreactors or on the membranes. Certainly, the microbes in bio-cake layers can degrade the rejected BMM and produce new BMM [8]. Generally, the quantity and quality of BMM are strongly dependent on their biodegradability. However, the biological fate of BMM and their complex interrelationships in MBRs remain unclear. This hinders a clear understanding of membrane fouling mechanisms.

Numerous studies regarding the biotransformation of BMM have been conducted. For instance, it has been reported that SMP and EPS could be reused for cell proliferation in case of substrate deficiency, though the reported biodegradability rates were low [9], [10]. Zhang et al. [11] stated that the EPS extracted from biofilm could be degraded by various microorganisms, including their own producers. Baker et al. [12] found that the large-size compounds in the residual dissolved organic matter (DOM) of wastewater treatment effluent were easily degraded under an aerobic condition, while the small-size fractions were readily degraded under an anaerobic condition. Thus, keeping a sufficient dissolved oxygen (DO) concentration in the bulk sludge can facilitate the elimination of large-size BMM, further improving the membrane filterability [13]. Recently, Okamura et al. [14] isolated and cultivated the microorganisms from activated sludge that could degrade large-size carbohydrates into small molecules. Yet these studies presented some insights into the fouling mechanisms of BMM from different aspects, many questions still remain unanswered.

Furthermore, there is limited information about the correlation between the biodegradation behaviors of BMM and the development of membrane fouling. Due to the heterogeneity of BMM, their biodegradation rates vary. It is, therefore, of interest to know which fractions are biologically removable, how their chemical constituents and properties change during the biodegradation process, and which fractions surely affect the membrane filtration. It is also interesting to track the origin of MSF from the biodegradability point of view. A fundamental research on the microbial transformation of BMM would be shed light on the membrane fouling of MBRs.

With the above in view, the objectives of this study are: (i) to understand the biodegradation behaviors of BMM through measurement of proteins (PN), polysaccharides (PS), and humic substances (HS) in a series of well-controlled bioassay tests, (ii) to assess the proportions of readily, slowly and non-biodegradable organics in the BMM sample through mathematical model simulating, (iii) to determine the changes of chemical characteristics and compositions of BMM during the biodegradation process from analysis of the molecule weight (MW) distributions and the parallel factor (PARAFAC) modeling of three-dimensional excitation-emission matrix (EEM) fluorescence spectra of BMM, and finally (iv) to obtain more knowledge about the biological control of MBR fouling.

## Experimental Section

### MBR operation and sample collection

All samples were collected from the membrane tank (aerobic condition) of a 50 L lab-scale MBR (see Figure S1) which treated complex synthetic wastewater (see Table S1). Two identical flat-sheet membrane modules (i.e., Modules A and B) (polyvinylidene fluoride, 0.1 µm, Sinap Corp., Shanghai, China) with the total surface area of 0.23 m^2^ (i.e., 0.115 m^2^ for each one) were submerged in the membrane tank. Modules A and B were operated with different membrane flux in different periods, respectively. The detailed operational conditions can be found elsewhere [15]. When the trans-membrane pressure (TMP) reached 25 kPa, the membrane modules were washed with high pressure water and then soaked in a 0.3% NaClO solution for ca. 12 hrs. Throughout the entire operation period, the SRT and hydraulic retention time (HRT) of the MBR were maintained at 20 days and 12–14 hrs, respectively.

The activated sludge mixed liquor of the membrane tank was first centrifuged in 50 mL tubes at 1280 g for 15 min. The supernatant in the centrifuge tubes was collected by filtering with filter papers (10 µm) and was regarded as the SMP sample. And the residual sludge pellets were re-suspended to the initial volume by adding appropriate amount of 0.05% NaCl solution. Afterwards, EPS were extracted by heating the mixed liquor to 60°C in a water bath for 30 min and then centrifuging it at 14940 g for 15 min, and finally filtering the supernatant with the filter papers [16]. To obtain representative data, the SMP and EPS samples were extracted at a regular interval, i.e., 1–2 weeks, and then the solution of the SMP/EPS extracted at different times was mixed for later use in the bioassay tests. Regarding the MSF samples, the fouled membrane module A (membrane flux: 26.1 L/(m^2^·h)) were taken out and flushed with high-pressure water to remove the bio-cake layers on membranes when the TMP reached 25 kPa. About 3000–4000 mL washed liquid was taken and then stirred using a glass rod to disperse the agglomerated sludge flocs or gel-like substances. The supernatant of washed liquid was further filtered with the filter papers and was regarded as the MSF samples. The particulate or colloidal organics (>0.45 µm) are considered as significant constituents in the sludge supernatant due to their significance in membrane fouling. Therefore, in order to take this fraction into account, all samples were filtered through 10 µm filter paper only. In this study, the SMP also included the particulate or colloidal organics. A tangential flow filtration system (Cogent™, Millipore Corporation, USA) equipped with a polyvinylidene fluoride (PVDF) pellicon cassette filter (1 kDa) was used to concentrate the samples and remove salts from them. All samples were then kept frozen at about −20°C for the bioassay tests accordingly.

### Bioassay protocols

The biodegradability assays of SMP, EPS, and MSF were performed in three triangular flasks for 21 days at 20°C under a dark condition, respectively. The experimental illustration of the bioassay tests are shown in Figure S2. Prior to the experiments, chemical oxygen demand (COD), total nitrogen (TN), and total phosphorus (TP) of the SMP, EPS, and MSF samples were determined according to the Chinese NEPA Standard Methods [17]. Subsequently, these samples were diluted with pure water to 1000 mL for keeping similar initial organic loading rates (approx. 120 mg COD/L), respectively. In order to avoid limits to microbial activity of inorganic nutrients (i.e., N, P) and the impacts of different nutrient concentrations on the bioassay testing results, TN and TP concentrations in all test solutions were kept at similar levels by adding nitrate and phosphate, respectively. The origins and compositions of the raw test solutions used for the bioassays were measured and summarized in Table 1. Afterwards, 3–5 mL of activated sludge taken from the membrane tank of the lab-scale MBR was applied as the microbial inoculums for the bioassay tests. The initial biomass concentrations were controlled at a very low level (<30 mg/L) so as to minimize the generation of microbial products resulted from microbial growth and decay [11] as well as reduce the adsorption of target compounds onto the sludge. Previously saturated air with water vapor was provided to maintain the DO concentration above 5 mg/L. To keep the biomass in suspension, the solutions in bioassay bottles were gently stirred using magnetic stirrers. Furthermore, in order to secure a high microbial activity during the course of bioassay tests, 0.5 mL of trace elements (FeSO_4_·7H_2_O 2.50 mg/L, CoCl_2_·6H_2_O 0.13 mg/L, NiCl_2_·6H_2_O 0.04 mg/L, CuSO_4_ 0.06 mg/L, H_3_BO_3_ 0.06 mg/L, ZnCl_2_ 0.06 mg/L, NaMoO_4_·2H_2_O 0.19 mg/L, MnSO_4_·4H_2_O 0.06 mg/L, CaCl_2_ 0.44 mg/L, MgCl_2_ 0.19 mg/L) was added into the three triangular flasks, respectively.

**Table 1:** Origin, concentration, and composition of initial solutions before the tests.

	SMP	MSF	EPS
Polysaccharides (mg/L)	21.37±1.75	27.97±3.73	19.14±1.09
Proteins (mg/L)	12.32±0.76	12.42±1.60	15.97±0.00
Chemical oxygen demand (mg/L)	122.64±10.99	120.60±5.49	108.55±3.12
Total nitrogen (mg/L)	11.57±4.99	9.57±7.26	8.85±0.12
Total phosphorus (mg/L)	4.13±1.50	2.02±1.69	5.47±0.00

Variations in the replicates (n = 3) are described as the average ±SD.

During the bioassays tests, 10 mL of samples were regularly taken out from each triangular flask at the time intervals of 0, 0.17, 0.33, 0.5, 1, 2, 3, 4, 6, 8, 10, 12, 14, 16, 18, and 21 d. PS and PN were quantified according to Dubois [18] and Lowry [19] methods, respectively. The influence of nitrate and nitrite on polysaccharide measurements was corrected by that of Drews [13]. Actually, the concentrations of nitrate and nitrite were so low that their impacts could be ignored. Moreover, HS were determined from their ultraviolet absorbance at 254 nm (UV_254_) (UNICO, UV-2000, USA). Here, UV_254_ was used to indicate the presence of humic substances in solutions qualitatively. The bioassay tests were conducted in triplication to obtain respective average values and standard deviations.

### Kinetic models for biodegradation of BMM

A first-order kinetic model (see Eq. 1) can be used to describe the biodegradation kinetics of organic matter. However, the fact that the BMM comprise of thousands of compounds with various degrees of biodegradability making the first-order kinetic model inadequate to describe the biodegradation process [20]. The multi-exponential G models (see Eq. 2) could be considered as alternative kinetic models, because they can not only deal with several pools of organic matter with different biodegradation potentials, but also describe the biodegradation kinetics of organic matter well [21], [22]. However, increased number of parameters due to increased organic pools also makes them difficult to apply. Therefore, the G models with only two or three components are usually applied (Eqs. 3 and 4).
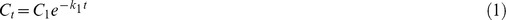


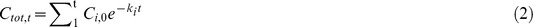


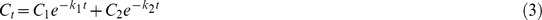


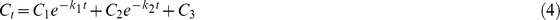



Although the G models with two pools (see Eq. 3) are more commonly employed to describe the biodegradation process [23], [24]. Yavich et al. [25] suggested that addition of a non-biodegradable component into the G models (see Eq. 4) could make it more adequate for simulating the biodegradation kinetics of organic matter, based on an analysis of numerous biodegradation curves. Gruenheid et al. [20] actually applied the G models with three pools successfully in describing the impact of temperature on the biodegradation of organic matter in soils.

Given the complexity of BMM in MBRs, the multi-exponential G model with three pools (see Eq. 5) was thus adopted in this study. In this model, the BMM were divided into three fractions: readily biodegradable BMM (BMM-*rd*), slowly biodegradable BMM (BMM-*sd*) and non-biodegradable BMM (BMM-*nd*).
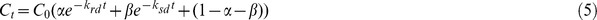
where, C*_t_* is the concentration of BMM at time *t*; C_0_ is the initial concentration of BMM; 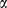 is the proportion of BMM-*rd*; *k_rd_* is the first-order rate constant of BMM-*rd*; 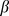 is the proportion of BMM-*sd*; *k_sd_* is the first-order rate constant of BMM-*sd*; and 1-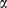-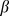 is the proportion of BMM-*nd*.

### Chemical analysis of BMM

To understand changes of the MW distributions of BMM during the bioassays, 50 mL liquor taken from each triangular flask at time zero, on days 12 and 21 was subjected to sample filtration with the filter papers. Then, the filtrate was subsequently filtered into a stirred dead-end membrane filtration cell (MSC300, Mosu corpopration, Shanghai, China) installed with a series of polyethersulfone membranes (0.45 µm, 100 kDa, 30 kDa, 5 kDa) to differentiate five fractions: >0.45 µm, 0.45 µm-100 kDa, 100–30 kDa, 30–5 kDa, <5 kDa. PS, PN, and HS in each fraction were determined, respectively, according to the methods mentioned above.

The EEM spectra of BBM were analyzed by using a fluorescence spectrophotometer (F-4500, Hitachi, Japan). The instrumental operation parameters followed that of Huang et al. [26]. The EEM data were preprocessed with the software Matlab7.0 (Math Works Inc., USA) to minimize the impact of other attributes on the EEM profiles. The blank EEM (Milli-Q water) was subtracted from each sample EEM. Measurements at excitation wavelengths below 220 nm and the emission wavelengths below 280 nm were excluded due to their invalidation.

The PARAFAC modeling of EEMs data was then conducted using Matlab7.0 with the DOMFluor toolbox (www.models.life.ku.dk). The three-way PARAFAC analyses are typically applied for the identification of DOM fluorescence components [27], [28]. The algorithm can decompose a series of EEMs data into three matrices (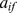, b*_jf_*, c*_kf_*) and a residual array (e*_ijk_*), thereby generating a trilinear equation (Eq. 6) that can minimize the sum of squared residuals and reflect the true underlying EEM spectra [29].
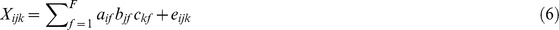


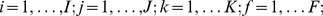
where, X*_ijk_* is the fluorescence intensity of sample *i* at the excitation wavelength *j* and the emission wavelength *k*; *F* represents the number of unique spectral profiles (components); 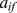
is the score matrix which is directly proportional to the concentration of the *f* ^th^ fluorophores; b*_jf_* and c*_kf_* are loading matrices of the excitation and emission spectrum of the *f* ^th^ fluorophores, respectively; and e*_ijk_* is the error matrix. A non-negativity constraint was applied to the equation to provide chemically meaningful results. Detailed PARAFAC modeling protocols can be found in the literature [30].

## Results

### Biodegradation of SMP, EPS, and MSF

**Figure 1:**
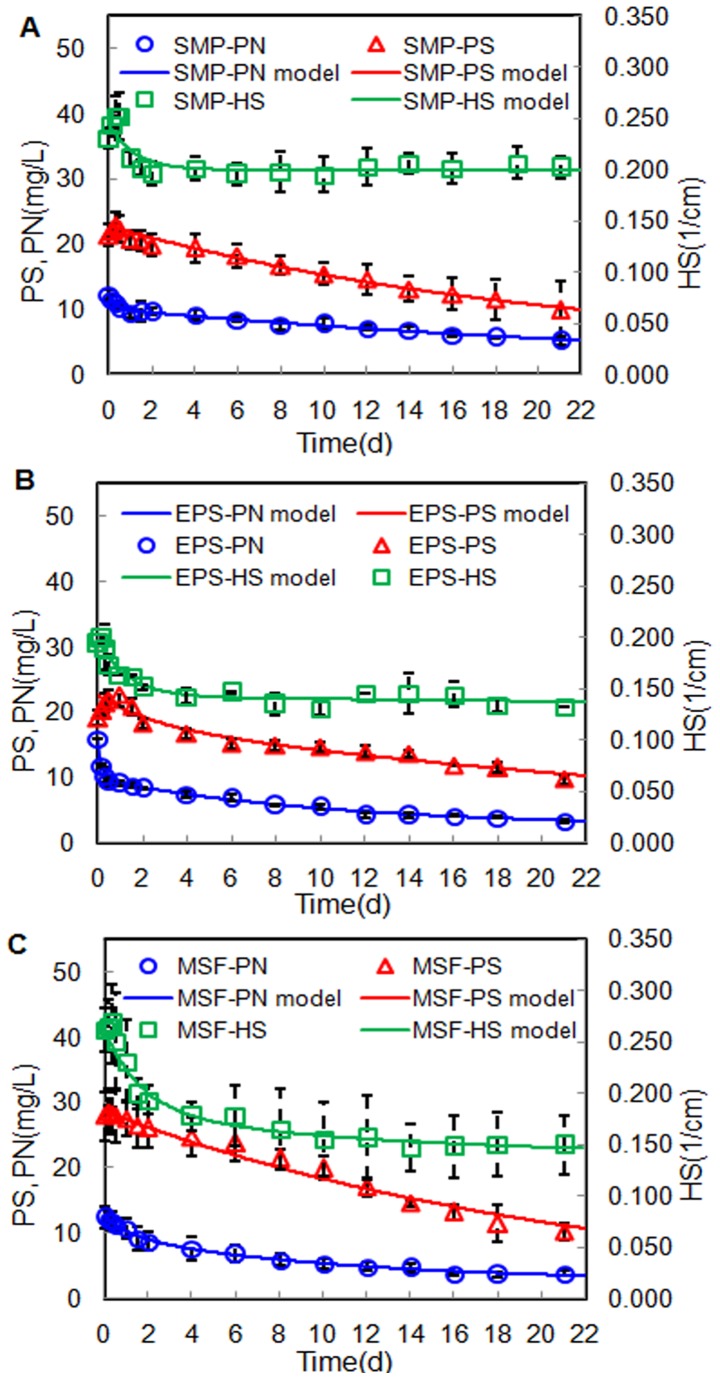
Measured data and modeling of BMM biodegradation in the 21-day bioassay tests.

The variations of PN, PS, and HS in the BMM during the biodegradation process are shown in Figure 1. The PS in SMP (SMP-PS) degraded slowly from 21.4 to 9.83 mg/L after a 21-day bioassay test (see in Figure 1A). In comparison, the PN in SMP (SMP-PN) underwent a rapid reduction from 12.3 to 9.60 mg/L within the first two days, but subsequently their degradation rates slowed down. In addition, the HS in SMP (SMP-HS) were only removed by 13% in the whole bioassay test; that is, over 80.0% of SMP-HS seemed to be non-biodegradable. It is interesting to note that the SMP-HS even presented a slightly increase from 0.195 (on day 6) to 0.206 cm^−1^(on day 21), indicating that some new HS were produced during the biodegradation process. As shown in Figure 1B, the PN in EPS (EPS-PN) were subject to much more rapid degradation from 16.0 to 8.33 mg/L within the first two days. Accordingly, the HS in EPS (EPS-HS) rapidly decreased by about 20% (from 0.194 to 0.152 cm^−1^) on the second day. This indicated that the EPS contained a great amount of readily biodegradable and unsaturated compounds (i.e., aromatic PN). But the PS in EPS (EPS-PS) had a rapid increase within the first 12 hrs and subsequently decreased in the following biodegradation period. In Figure 1C, it was found that MSF had a PS concentration similar to that of SMP and their biodegradation behaviors were also quite analogous. Finally, an average removal rate of 63.6% MSF-PS was achieved after the 21-day incubation. Moreover, approximately 69.3% of MSF-PN and 43.0% of MSF-HS were removed.

### Kinetic modeling for biodegradation of SMP, EPS, and MSF

The compositions and degradation kinetic coefficients of the BMM obtained from the G models are summarized in Table 2. Except for the simulation of SMP-HS (*r* ^2^ = 0.75), the G models for other components were all in good agreement with respective measured data (*r* ^2^>0.94). The less accurate SMP-HS simulation was possibly due to the production of HS during the bioassay tests. As shown in Table 2, both SMP-PS and SMP-PN were mainly composed of slowly biodegradable matter (86.6 and 81.4%, respectively) with biodegradation rate constants of 0.048 and 0.030 d^−1^, respectively. Moreover, the majority of SMP-HS were non-biodegradable matter (81.4%). Menniti et al. [7] also reported that a large fraction of the SMP were very slowly degradable organic matter. Nonetheless, SMP-PN and SMP-HS were found to have the same lower proportions of readily biodegradable fractions (18.6%). The readily biodegradable SMP-HS were attributed to the presence of some easily biodegradable aromatic PN with a higher UV_254_ absorbance (see the section 3.4).

**Table 2:** Parameters describing the fit of degradation data by the multi componential G model.

BMM		BMM-*rd*		BMM-*sd*		BMM-*nd*	Kinetics Equations	*r^2^*
		Percent. (%)	*k_rd_* (d^−1^)	Percent. (%)	*k_sd_* (d^−1^)	Percent. (%)		
	Polysaccharides	-	-	86.6	0.048	13.4	C_t_ = 22.76 (86.6% e^−0.048t^+13.4%)	0.98
SMP	Proteins	18.6	2.994	81.4	0.030	-	C_t_ = 12.32 (81.4% e^−0.030t^+18.6% e^−2.994t^)	0.99
	Humic substances	18.6	0.842	-	-	81.4	C_t_ = 0.245 (18.6% e^−0.842t^+81.4%)	0.75
	Polysaccharides	-	-	65.8	0.190	34.2	C_t_ = 23.5 (65.8% e^−0.190t^+34.2%)	0.94
EPS	proteins	39.1	6.090	45.7	0.094	15.2	C_t_ = 15.97 (39.1% e^−6.090t^+45.7% e^−0.094t^+15.2%)	0.99
	Humic substances	27.7	0.761	72.3	0.002	-	C_t_ = 0.197 (27.7% e^−0.761t^+72.3% e^−0.002t^)	0.94
	Polysaccharides	-	-	100.0	0.045	-	C_t_ = 28.63 e^−0.0447t^	0.98
MSF	Proteins	22.6	0.760	57.0	0.092	20.4	C_t_ = 12.42 (22.6% e^−0.760t^+57.0% e^−0.092t^+20.4%)	0.99
	Humic substances	30.4	0.605	17.0	0.091	52.7	C_t_ = C_t_ = 0.266 (30.4% e^−0.605t^+17.0% e^−0.091t^+52.7%)	0.97

- Not possible to estimate; BMM-*rd*, BMM-*sd* and BMM-*nd* are the readily biodegradable, slowly biodegradable and non-biodegradable fraction in the BMM.

However, in the case of EPS, 39.1% of EPS-PN was identified as readily biodegradable matter with a higher degradation rate constant (6.1 d^−1^). Additionally, the proportions of the slowly biodegradable and non-biodegradable fractions of EPS-PN were determined to be 45.7 and 15.2%, respectively. In contrast, EPS-PS mainly consisted of slowly biodegradable matter (65.8%) and non-biodegradable matter (34.2%), respectively. Wang et al. [31] also showed that 50% of EPS-PS and 30% of EPS-PN produced by aerobic granules were biodegradable. Table 2 indicates that the degradation rate constants of slowly biodegradable fractions of EPS, including PS, PN, and HS, were all greater than that of SMP. These results suggest that the EPS were much more readily biodegradable than the SMP, which is in agreement with the findings of Menniti et al. [7]. Interestingly, it was found that MSF-PS were completely dominated by slowly biodegradable matter, with a degradation rate constant (0.045 d^−1^) close to that of SMP-PS (0.048 d^−1^). This corroborated the similar biodegradation behaviors of SMP-PS and MSF-PS mentioned above. This finding implies that MSF-PS could be mainly derived from the deposition/retention of SMP-PS. Meanwhile, the non-biodegradable fraction was not detected in MSF-PS, indicating that the non-biodegradable SMP-PS could be the small-MW compounds and hence passed through the membranes. The rate constant of the readily biodegradable SMP-PN (3.0 d^−1^) was much larger than that of the readily biodegradable MSF-PN (0.76 d^−1^), despite similar proportions (18.6 and 22.6%, respectively). Moreover, MSF-PN had a smaller proportion of slowly biodegradable fraction (57.0%) with a higher rate constant (0.092 d^−1^) which were both close to those of EPS (45.7% and 0.094 d^−1^). These phenomena suggest that the SMP-PN and MSF-PN had different chemical characteristics and compositions; on the contrary, EPS-PN were most likely to be an important origin of MSF-PN.

### Impacts of biotransformation on molecular size of BMM

**Figure 2:**
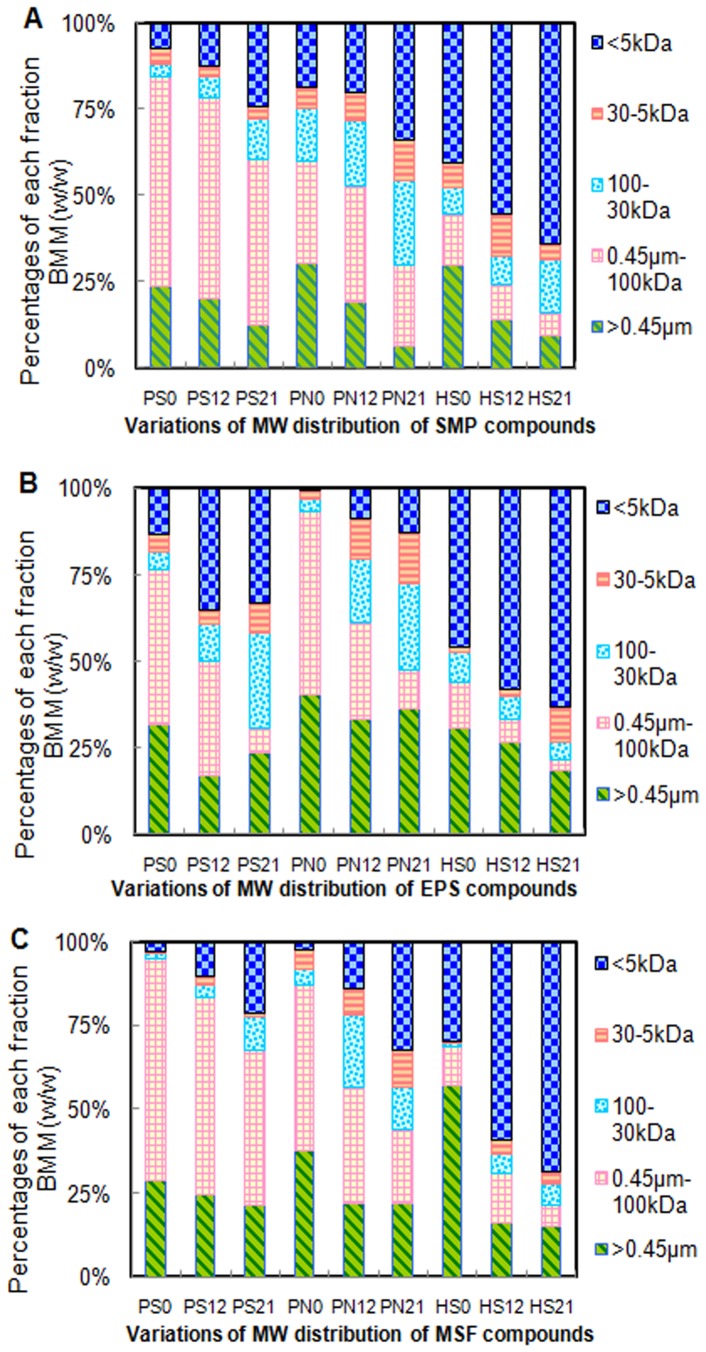
Variations of molecular weight (MW) distribution of BMM during the biodegradation process.

The MW distributions of SMP, EPS, and MSF were analyzed during the biodegradation process. As shown in Figure 2, the SMP, EPS, and MSF prior to bioassay tests were dominated by large-MW (>100 kDa) compounds, accounting for over 60.0% of total BMM. Particularly, both the large-MW MSF-PS and MSF-PN had a higher proportion up to 90.0% of the total BMM. Additionally, SMP and EPS contained a portion of small-MW compounds (<5 kDa) ranging from 10.0 to 20.0%. This is in good agreement with previous studies [32], [33]. Interestingly, all of the small-MW compounds (<5 kDa) in SMP, EPS, and MSF had a higher proportion of HS instead of PN or PS, indicating that the small-MW compounds were dominated by unsaturated carbon bonds, such as aromatic groups. Moreover, it was also found that a quite higher level of HS presented in the large-MW compounds (>100 kDa) in all BMM samples. This could be due to the colloids or aromatics-like proteins in the large-MW compounds disturbing the UV_254_ measurement by their UV_254_ absorption or light scattering.

After a biodegradation period of 21 days, the large-MW compounds (>0.45 µm and 0.45 µm-100 kDa) in the SMP, EPS, and MSF were all subject to an obvious reduction. The percentages of colloidal SMP-PN and SMP-PS (>0.45 µm) decreased by over 50.0 and 80.0%, respectively (Figure 2A). The EPS-PN in the 0.45 µm-100 kDa fraction dropped sharply from 44.8 to 7.20%. Figure 2C shows that the percentages of large-MW PS and PN (>100 kDa) in the MSF also dropped by about 30.0 and 50.0%, respectively. On the contrary, the proportions of small-MW compounds (<5 kDa) in the SMP, EPS and MSF increased considerably during the biodegradation process. The MSF-PS and MSF-PN of the <5 kDa fraction increased from 2.8 and 2.5 to 21.1 and 32.5%, respectively. Similarly, the small-MW compounds (<5 kDa) in the EPS became a dominant component at the end of the bioassay tests. The proportions of the small-MW EPS-PS and EPS-PN reached 33.7 and 13.2% after the 21-day bioassay, respectively. Additionally, HS followed similar trends that the large-MW compounds decreased while the small-MW compounds increased.

### Microbial transformation of fluorescent components in BMM

**Figure 3:**
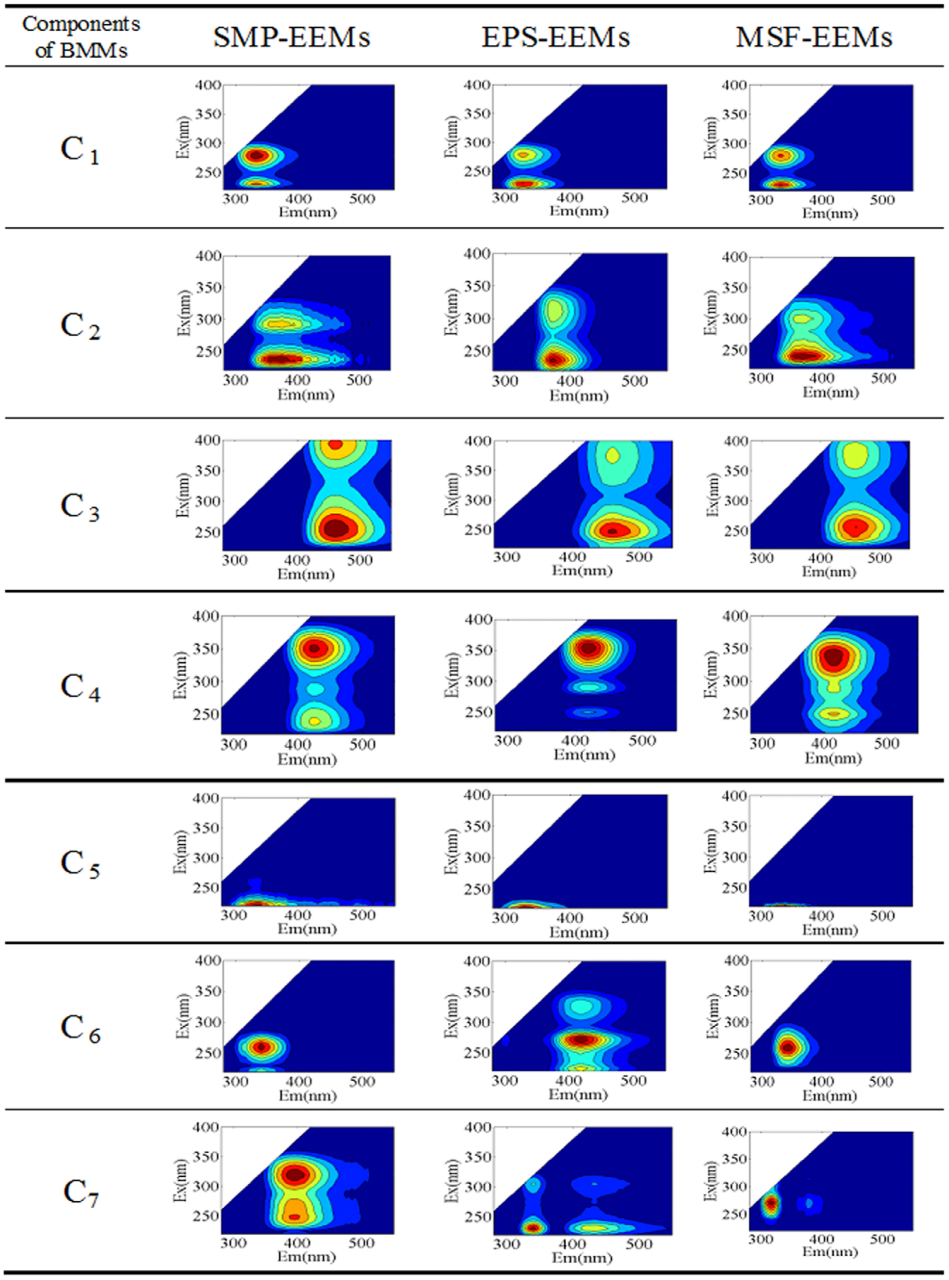
Contour plots of the seven components identified from the SMP-EEMs, EPS-EEMs and MSF-EEMs dataset.

In this study, the EEM spectra of the SMP, EPS and MSF compounds (SMP-EEMs, EPS-EEMs and MSF-EEMs) at time zero, and on days 2, 8, 14 and 21 were analyzed by the PARAFAC model. Seven components were included in the model simulation for SMP-EEMs, EPS-EEMs and MSF-EEMs, which were computed on the basis of the minimum spectral sum of squared error (see in Figure S3). Identification of each of them followed previous EEM-PARAFAC studies [28], [34] and typical fluorescence regional assignments [35]. Contour plots of the seven components in the SMP-EEMs, EPS-EEMs, and MSF-EEMs dataset are shown in Figure 3, respectively. Their line plots and the descriptions are summarized in Table S2.

Obviously, the SMP, EPS, and MSF had five similar components (C_1_, C_2_, C_3_, C_4_ and C_5_) in common but have two other different components (C_6_ and C_7_), indicating the complex relationships among SMP, EPS, and MSF. The components of C_1_ and C_5_ were typically identified as tryptophan-like PN, as described elsewhere [27]: C_1_ comprised two peaks with an excitation maximum at 230 (Peak T_1_) and 280 nm (Peak T_2_) with 330 nm emission, as reported by Wu et al. [36]; C_5_ occurred at 220 nm excitation with 340 nm emission, which is similar to the traditional Peak T_2_ (Ex/Em, 225–237/340–381 nm). The components of C_2_, C_3_, and C_4_ were related to the HS fluorophores [27]: C_2_ with an emission maximum at 230–240 nm and 290–300 nm with 370 nm emission is typically defined as the microbial HS fluorophores [37], [38]; C_3_ is due to the traditional terrestrial HS fluorophores [39], [40] which could be derived from the feed wastewater; C_4_ has not been traditionally defined, though it is only present in a wastewater system [41]. Murphy et al. [40] and Stedmon et al. [42] reported that C_4_ represented the HS fluorophores produced from microbial degradation and could be an indicator of the level of nutrient enrichment in wastewater. In addition to these similarities, the PARAFAC components of SMP, EPS, and MSF showed some differences: 1) C_6_ in MSF-EEMs were similar to C_6_ in SMP-EEMs which was located at a similar emission spectrum to the typical tryptophan-like PN (Ex/Em, 275/340 nm, Peak T_1_), whereas C_6_ in EPS-EEMs was another microbial HS fluorophores component [40] and it was also present in SMP-EEMs; 2) C_7_ in MSF-EEMs was associated with free amino acids [38], [39], [40] which was also found in EPS-EEMs.

**Figure 4:**
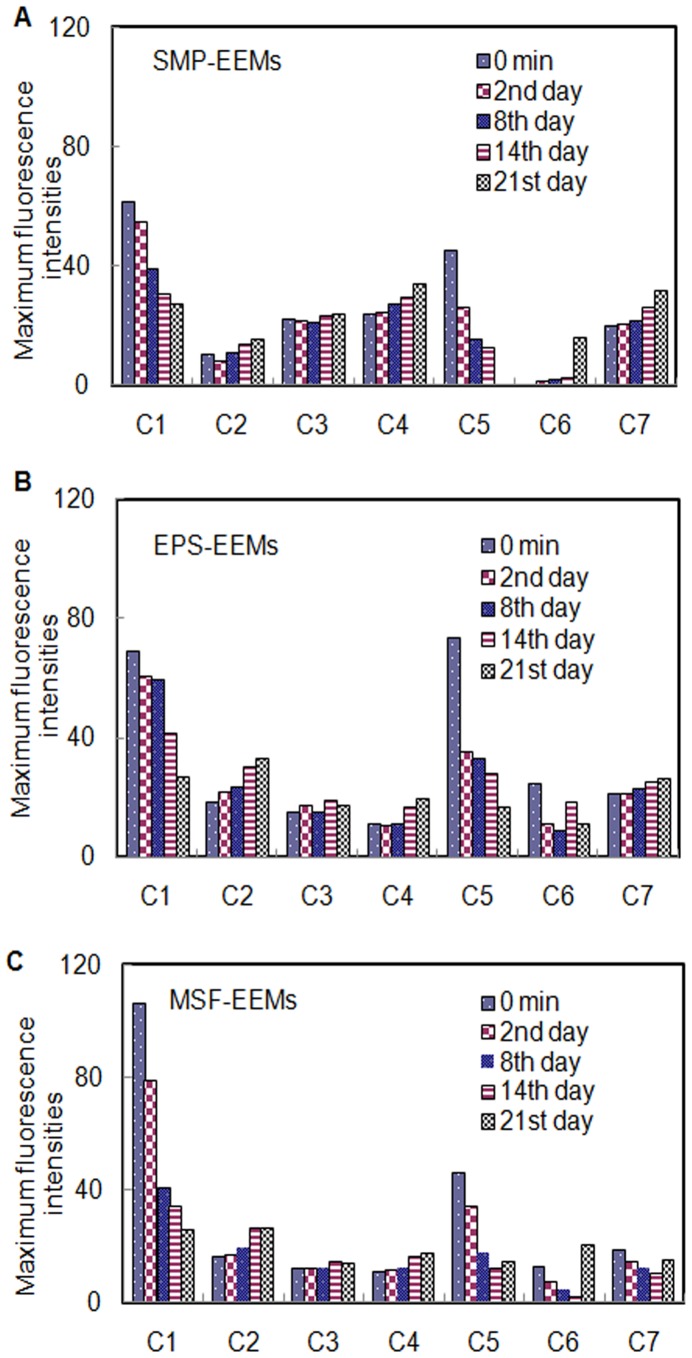
Microbial transformation of the maximum fluorescence intensities (F_max_) of the PARAFAC components in the SMP-EEMs, EPS-EEMs and MSF-EEMs.

The variations of the maximum fluorescence intensities (F_max_) of the PARAFAC components in SMP, EPS, and MSF through the biodegradation process are shown in Figure 4. The raw solutions of SMP, EPS, and MSF were all dominated by C_1_ and C_5_; that is, the tryptophan-like PN were the major fluorescent components in all of the BMM. Regarding the MSF samples, the PN components (C_1_, C_5_, and C_7_ in MSF) accounted for 77.0% of the total F_max_, further confirming that the fluorescent components in MSF were mainly composed of large-MW PN rather than HS. On the other hand, it indicates that the HS consisted of small-MW components that were not retained by membranes, corresponding to the results presented in the section 3.3. Both F_max_ of C_1_ and C_5_ in all BMM compounds were subject to an apparent degradation, while that of the microbial HS components (C_2_, C_4_) showed an obvious increase. This suggested that the non-humics in SMP, EPS and MSF all underwent a microbial humification process. Some of the large-MW compounds were transformed into the small-MW HS, which could further corroborate the results detailed in the section 3.3. This further indicated that the PN compounds were much more readily biodegradable than the HS. The F_max_ of C_3_ representing the terrestrial HS fluorophores remained at a stable level, indicating that the component of C_3_ likely derived from the feed wastewater and was refractory. More interestingly, the terrestrial HS was found in all BMM, suggesting that besides the microbial origins, the residual and refractory compounds contained in the feed wastewater could be contributed by BMM. Among all BMM, the amount of the HS in the feed wastewater was not so much as revealed by their F_max_. Our previous work found that the chemical natures and compositions of these compounds in the feed wastewater were quite different from those of SMP [43]. Although the remaining terrestrial and man-made organics were not of microbial origins, they cannot be excluded out from SMP.

Due to the different PARAFAC components (i.e., C_6_ and C_7_) in SMP, EPS and MSF, the changes of F_max_ varied significantly. For example, although the C_6_ in SMP was similar to that in MSF, it had different biodegradation potential: the F_max_ of C_6_ in SMP increased slightly; however, the F_max_ of C_6_ in MSF showed an obvious decrease. The F_max_ of C_6_ in MSF on day 21 increased suddenly, likely resulting from a random error due to the weak F_max_ which was difficult in detecting adequately. Moreover, the C6 in EPS and C7 in SMP had similar spectral features, as identified as the microbial HS, but their F_max_ changes were different. The C_6_ in EPS seemed to be more liable to biodegradation and showed higher biodegradation potential.

## Discussion

### Homology of MSF and SMP/EPS

Based on the experimental measurements and mathematical model simulation, we can make a plausible hypothesis that MSF-PS and SMP-PS were somewhat homologous whereas MSF-PN were likely derived from EPS-PN. In fact, both MSF-PS and SMP-PS had similar MW distributions, and most of which were composed of large-MW compounds (>100 kDa). This helps to explain why both SMP-PS and MSF-PS had similar biological fates. During the MBR operation, almost all of SMP-PS were retained on the membranes due to their large molecular size. Owing to the small MW, SMP-PN, on the contrary, were transported through the membranes and appeared in the effluent. In our previous studies, the rejection degree of SMP-PS was found to be almost twice as high as that of SMP-PN [44], [45]. In addition, the EPS released by the microbes in the bio-cake layers appeared to have a much larger PN/PS ratio [43]; therefore, PN were one major constituent of EPS. As a consequence, EPS-PN could be one of the major origins of MSF-PN. Generally, these results support the following hypothesis: 1) MSF-PS could be mainly derived from SMP-PS due to the large MW and low biodegradability; 2) MSF-PN could be mainly derived from EPS-PN, which may be released by microbes in the bio-cake layers or gel layers.

### Hydrolysis and humification of large-MW compounds

The analysis of the MW distributions of BMM in this work shows that the large-MW compounds had a faster bacterial utilization rate than did the small-MW counterparts. Amon et al. [46] and Barker et al. [12] also observed such a phenomenon, pointing out that large-MW PN were more readily biodegradable than large-MW PS. In the bioassay tests, the levels of small-MW compounds gradually increased, indicating that the small-MW compounds were either more refractory to biodegradation or more prone to be generated as a type of biodegradation products. In fact, the large-MW compounds could not be directly utilized by microorganisms; they need to be hydrolyzed into small molecules before being consumed. Therefore, hydrolysis can be viewed as an important removal pathway of the large-MW compounds. The increase of small-MW PS and PN during the biodegradation process, as shown in Figure 2, can reveal such a pathway. Moreover, a slight increase in the F_max_ of C_6_ (i.e., amino acids) in SMP demonstrated that some of the PN were hydrolyzed to free amino acids. On the other hand, the humification could be considered as another important mechanism responsible for the microbial transformation of BMM. The obvious increase of HS (i.e., C_2_, C_4_) in the bioassay tests suggested that a great number of BMM underwent a humification continuum from the large-MW compounds to the small-MW and refractory HS. Furthermore, the production of the new HS could lead to an underestimation of the net loss of HS. This further explains the poor fit of HS originating from SMP in the multi-exponential G models. The fact that Namour and Muller [24] also found the HS increased by 40% after a 21-day bioassay test corroborates the humification process of BMM.

### Concluding remarks

Our work reported the microbial transformation of SMP, EPS, and MSF collected from a lab-scale MBR. The results showed that the biodegradation behaviors of these BMM were different from each other, presumably due to their different chemical compositions as well as their different origins. The biodegradation kinetics and the compositions in terms of biodegradability of SMP, EPS, and MSF were described well by the G models. It was found that slowly biodegradable PS dominated SMP and MSF. The non-biodegradable PS and readily biodegradable PN accounted for a larger part of EPS. Due to the slow biodegradation rate and large-MW of SMP-PS, enhancement of SMP-PS biodegradability is one of the main avenues to alleviate membrane fouling. Given that EPS-PN being one of the main sources of MSF-PN, controlling the production and release of EPS compounds could be of help in reducing membrane fouling. The results of EEM-PARAFAC model of BMM showed that humification and hydrolysis could be viewed as two important biotransformation mechanisms of large-MW compounds. In general, the results obtained from this study can not only improve our understanding of the fouling mechanisms in MBRs, but are also useful for understanding the biological fate of BMM.

## Supporting Information

Figure S1 **The configuration of the bench-scale MBR.**
(TIF)

Figure S2 **The schematic diagram of bioassay tests.**
(TIF)

Figure S3 **Residual comparison of components 6–8 of SMP-EEMs (a), EPS-EEMs(b), and MSF-EEMs (c) identified with DOMFluor-PARAFAC model.**
(DOC)

Table S1 **Compositions of synthetic wastewater.**
(DOC)

Table S2 **The line plots and the descriptions of the seven components in the SMP-EEMs, EPS-EEMs and MSF-EEMs dataset.**
(DOC)
