# Supplementary figures and images for: Microbial Transformation of Biomacromolecules in a Membrane Bioreactor: Implications for Membrane Fouling Investigation
Source: PLoS One. 2012 Aug 9;7(8):e42270. doi: 10.1371/journal.pone.0042270 (PMC3415425; doi:10.1371/journal.pone.0042270)

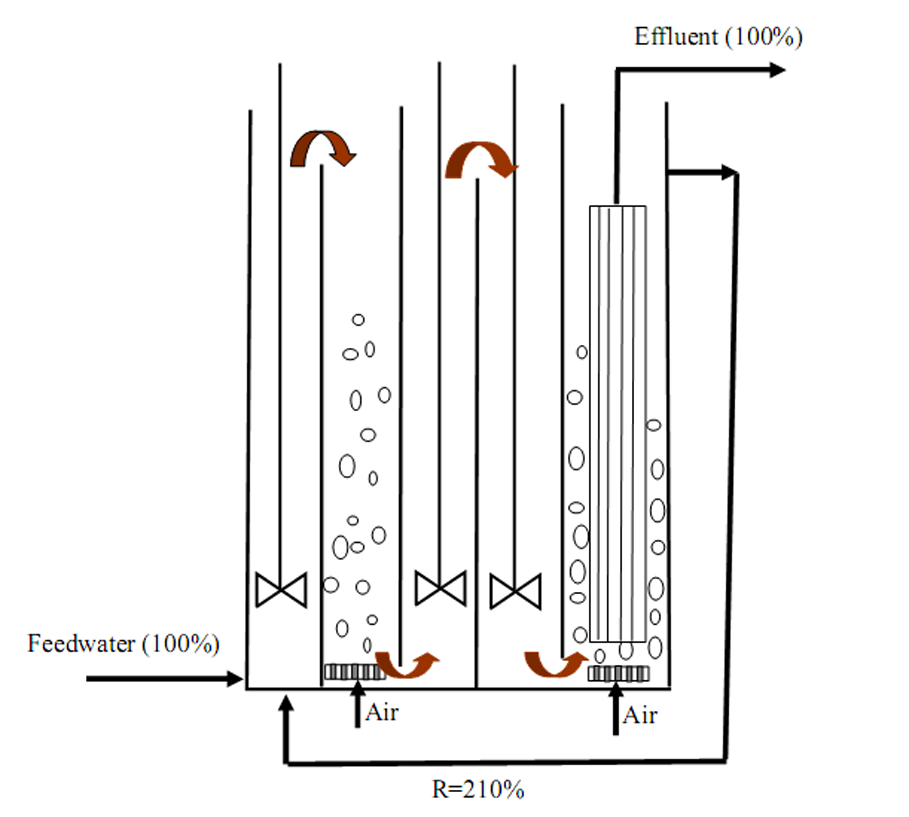

Supplement: Figure S1 — The configuration of the bench-scale MBR. (TIF) [file pone.0042270.s001.tif]

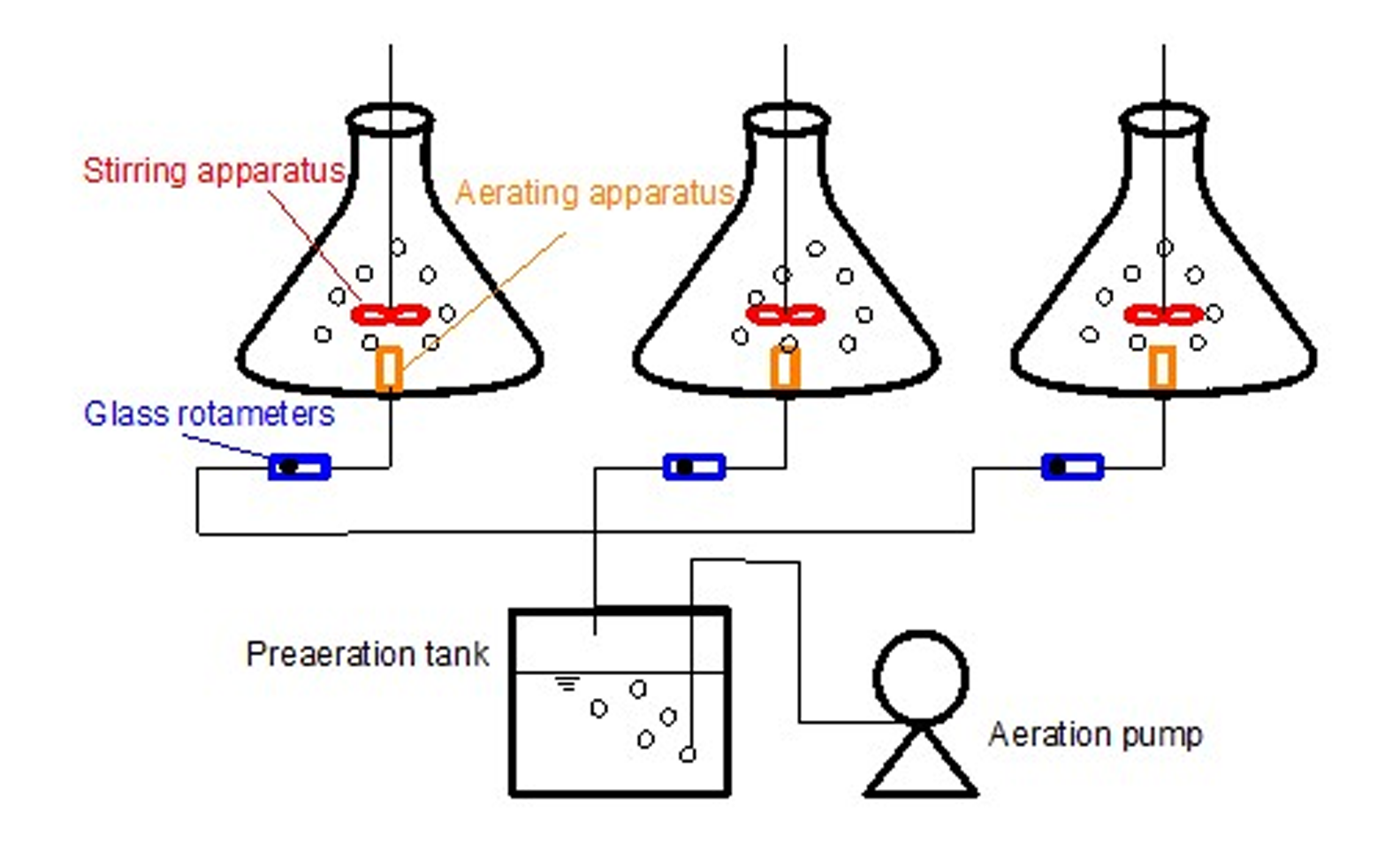

Supplement: Figure S2 — The schematic diagram of bioassay tests. (TIF) [file pone.0042270.s002.tif]
