# Supplementary material for: Microbial Transformation of Biomacromolecules in a Membrane Bioreactor: Implications for Membrane Fouling Investigation
Source: PLoS One. 2012 Aug 9;7(8):e42270. doi: 10.1371/journal.pone.0042270 (PMC3415425; doi:10.1371/journal.pone.0042270)
Supplement: Figure S3 — Residual comparison of components 6–8 of SMP-EEMs (a), EPS-EEMs(b), and MSF-EEMs (c) identified with DOMFluor-PARAFAC model. (DOC) [file pone.0042270.s003.doc]

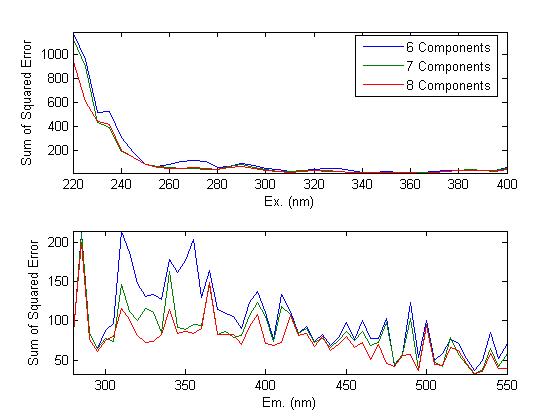

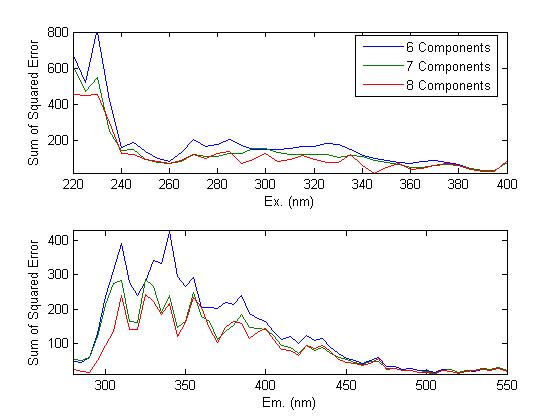

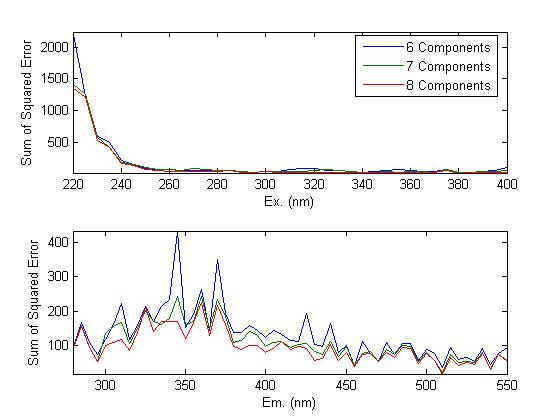


(a)

(b)

(c)

Figure S3. Residual comparison of components 6–8 of SMP-EEMs (a), EPS-EEMs (b), and MSF-EEMs (c) identified with DOMFluor-PARAFAC model. It can be seen that 7 components are enough for the PARAFAC modeling of the EEM spectra.

In fact, the PARAFAC model consisting of nine components was also computed. But its spectral sum of squared error began to be over that of the PARAFAC model consisting of seven or eight components. Therefore, the number of component was thought to be eight or seven, whose spectral sum of squared error was similar and smaller than that of the PARAFAC model consisting of six components. However, when the PARAFAC model consisting of eight components was computed, there always showed some strange and unreasonable components so that they could be not identified according to previous EEM-PARAFAC studies and typical fluorescence regional assignments. So, the PARAFAC model consisting of seven components were selected and computed.
