# Supplementary material for: Microbial Transformation of Biomacromolecules in a Membrane Bioreactor: Implications for Membrane Fouling Investigation
Source: PLoS One. 2012 Aug 9;7(8):e42270. doi: 10.1371/journal.pone.0042270 (PMC3415425; doi:10.1371/journal.pone.0042270)
Supplement: Table S1 — Compositions of synthetic wastewater. (DOC) [file pone.0042270.s004.doc]

Table S1. Compositions of synthetic wastewater.

| Ingredient | mg/L | Trace elements | mg/L |
| --- | --- | --- | --- |
| Na-acetate | 35 | FeSO_4_.7H_2_O | 2.50 |
| KH_2_PO_4_ | 23 | ZnCl_2_ | 0.06 |
| K_2_HPO_4_ | 28 | MnCl_2_.4H_2_O | 0.06 |
| NH_4_Cl | 80 | NaMoO_4_ .2H_2_O | 0.19 |
| Starch | 162 | CoCl_2_.6H_2_O | 0.13 |
| Milk powder | 200 | NiCl_2_.6H_2_O | 0.04 |
| Sucrose | 141 | CuSO_4_ | 0.06 |
| Urea | 50 | CaCl_2_ | 0.44 |
| Peptone | 32 | H_3_BO_3_ | 0.06 |
| Yeast extract | 60 | MgCl_2_ | 0.19 |
| Beef extract | 60 |  |  |
| NaHCO_3_  Na_2_CO_3_ | 80  80 |  |  |
