# Supplementary material for: Microbial Transformation of Biomacromolecules in a Membrane Bioreactor: Implications for Membrane Fouling Investigation
Source: PLoS One. 2012 Aug 9;7(8):e42270. doi: 10.1371/journal.pone.0042270 (PMC3415425; doi:10.1371/journal.pone.0042270)
Supplement: Table S2 — The line plots and the descriptions of the seven components in the SMP-EEMs, EPS-EEMs and MSF-EEMs dataset. (DOC) [file pone.0042270.s005.doc]

Table S2 (a). Contour plots and line plots of the seven components identified from the SMP-EEMs dataset. Dot lines and real lines show the excitation and the emission loadings of the whole dataset. The spectral characteristics (locations and sources) of the seven components identified from PARAFAC modeling of SMP.

| Components  of SMP | The contour plots | The line plots | The spectral characteristics (References) |
| --- | --- | --- | --- |
| C _1_ | 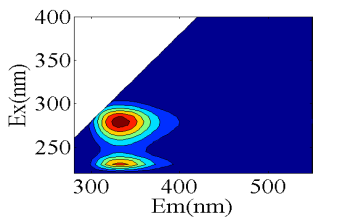 | 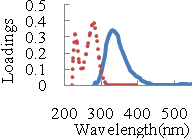 | Protein, tryptophan-like Ex230(280)/Em330  (Hudson et al., 2007; Wu et al., 2011) |
| C _2_ | 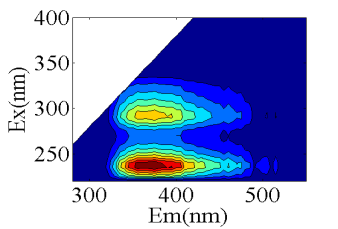 | 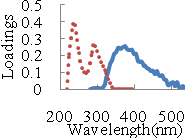 | Microbial humic-like  Ex235(290)/Em375  (Yamashita et al., 2008; Yamashita et al., 2008) |
| C _3_ | 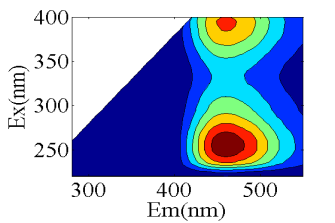 | 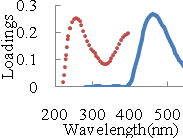 | Terrestrial humic-like in high nutrient and wastewater  Ex255(395)/Em460  (Baghoth et al., 2011; Murphy et al., 2011) |
| C _4_ | 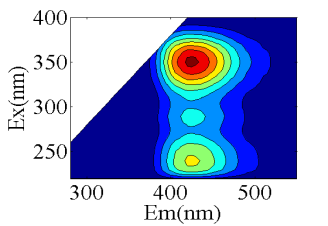 | 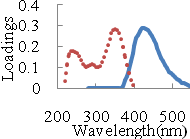 | Microbial humic-like  Ex350(240)/Em415  (Murphy et al., 2011; Stedmon et al., 2005; Wu et al., 2011; Yu et al., 2010) |
| C _5_ | 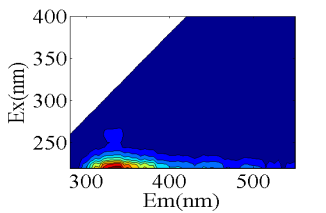 | 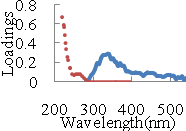 | Protein, tryptophan-like  Ex220/Em340  (Hudson et al., 2007; Murphy et al., 2011) |
| C _6_ | 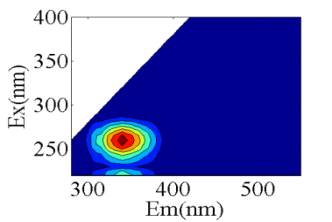 | 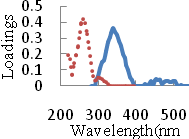 | Protein, tryptophan-like  Ex260/Em340  (Hudson et al., 2007; Murphy et al., 2011) |
| C_7_ | 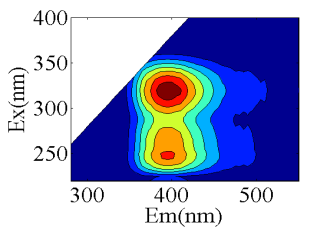 | 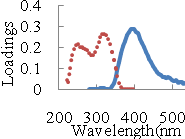 | Microbial humic-like  Ex320(250)/Em395  (Murphy et al., 2011) |

Table S2 (b). Contour plots and line plots of the seven components identified from the EPS-EEMs dataset. Dot lines show split-half validations of excitation and the emission loadings are real lines. The spectral characteristics (locations and sources) of the seven components identified from PARAFAC modeling of EPS

| Components  of EPS | The contour plots | The line plots | The spectral characteristics (References) |
| --- | --- | --- | --- |
| C_1_ | 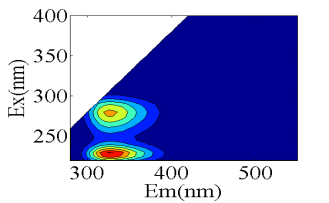 | 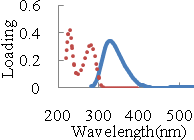 | Protein, tryptophan-like Ex230(280)/Em325  (Wu et al.,2011;  Hudson et al.,2007) |
| C_2_ | 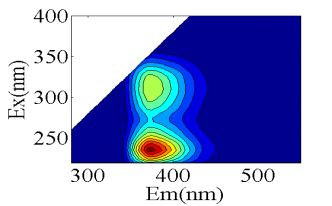 | 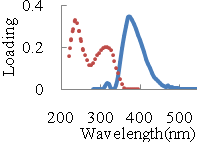 | Microbial humic-like  Ex235(310)/Em375  (Yamashita et al., 2008a,b) |
| C_3_ | 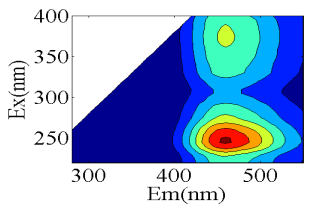 | 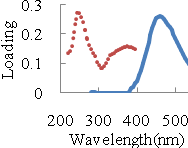 | Terrestrial humic-like in high nutrient and wastewater Ex245(375)/Em460  (Baghoth et al., 2011;  Murphy et al., 2011) |
| C_4_ | 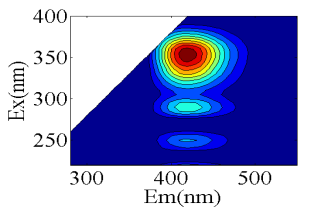 | 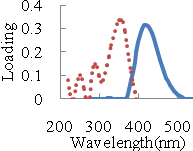 | Microbial humic-like  Ex350(240)/Em415  (Murphy et al., 2011;Yu et al., 2010; Stedmon et al, 2005; Wu et al.,2011) |
| C_5_ | 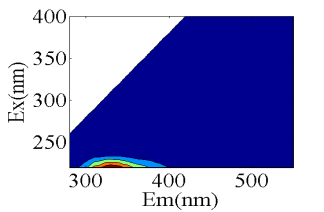 | 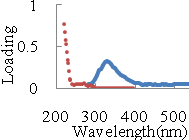 | Protein, tryptophan-like  Ex220/Em330  (Murphy et al., 2011;  Hudson et al., 2007) |
| C_6_ | 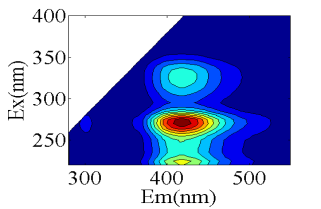 | 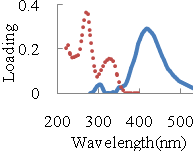 | Microbial humic-like  Ex270(225, 325)/Em 415  (Murphy et al., 2011; Yu et al., 2010; Stedmon et al, 2005; Wu et al.,2011) |
| C_7_ | 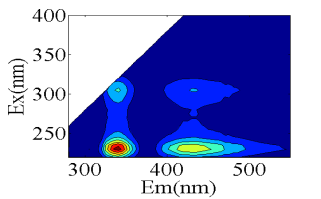 | 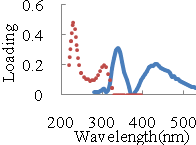 | Protein, Amino acids Ex230(305)/Em340(430)  (Baghoth et al., 2011;  Murphy et al., 2011) |

Table S2 (c). Contour plots and line plots of the seven components identified from the MSF-EEMs dataset. Dot lines show split-half validations of excitation and the emission loadings are real lines. The spectral characteristics (locations and sources) of the seven components identified from PARAFAC modeling of MSF.

| Components  of MSF | The contour plots | The line plots | | The locations and sources(References) | |
| --- | --- | --- | --- | --- | --- |
| C _1_ | 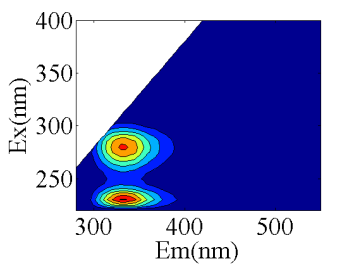 | 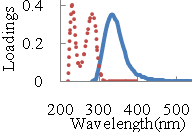 | | | Protein, tryptophan-like Ex230(280)/Em330  (Wu et al.,2011;  Hudson et al.,2007) |
| C _2_ | 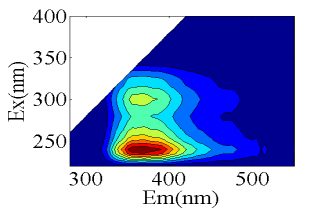 | 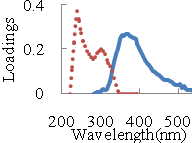 | Microbial humic-like  Ex240(300)/Em370  (Yamashita et al., 2008; Murphy et al., 2011) | | |
| C _3_ | 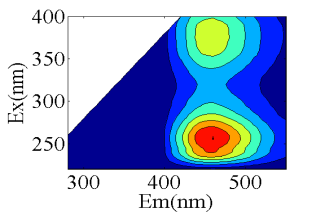 | 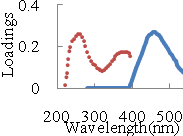 | Terrestrial humic-like  Ex255(375)/Em460  (Baghoth et al., 2011; Murphy et al., 2011) | | |
| C _4_ | 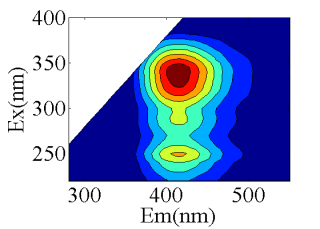 | 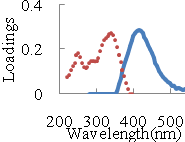 | Microbial humic-like /Wastewater  Ex350(240)/Em415  (Murphy et al., 2011;  Yu et al., 2010;  Stedmon et al, 2005) | | |
| C _5_ | 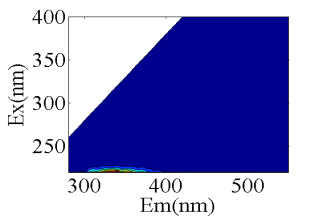 | 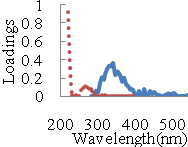 | Protein, tryptophan-like  Ex220/Em340  (Murphy et al., 2011; Hudson et al., 2007) | | |
| C _6_ | 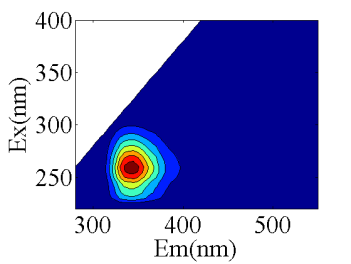 | 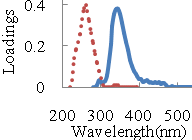 | Protein, tryptophan-like  Ex260/Em345  (Murphy et al., 2011; Hudson et al., 2007;) | | |
| C_7_ | 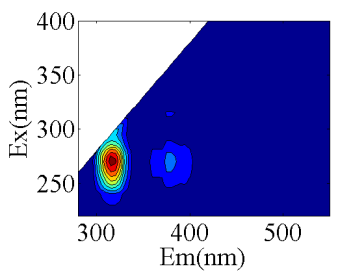 | 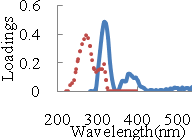 | Protein, Amino acids  Ex270/Em315(375)  (Baghoth et al., 2011; Murphy et al., 2011) | | |
